# Cardiovascular complications of pregnancy

**DOI:** 10.1172/JCI198808

**Published:** 2026-01-02

**Authors:** Yijun Yang, Jennifer Lewey, Zoltan Arany

**Affiliations:** 1 Cardiovascular Institute, and; 2 Division of Cardiology, University of Pennsylvania Perelman School of Medicine, Philadelphia, Pennsylvania, USA.

## Abstract

The maternal cardiovascular system undergoes dramatic remodeling in response to the stresses of pregnancy. Although in most cases these changes are temporary and well tolerated, in others they can give rise to complications, including cardiomyopathy, coronary artery disease, and hypertensive cardiovascular disease. Despite an increasing number of preclinical models to study these diseases, specific treatments for any of these pregnancy complications are lacking. As the maternal mortality rate is rising in the United States, it is critical to understand the molecular mechanisms driving cardiovascular changes during pregnancy, and the pathology that can result.

## Introduction

Pregnancy is accompanied by a series of physiological adaptations to sustain fetal growth while maintaining maternal homeostasis. The maternal heart undergoes physiological remodeling to cope with the increase in maternal blood volume and cardiac output (1–7). Cardiac wall thickness and cardiac mass increase in parallel without overt cardiac dysfunction (2–5, 8). In addition to these dramatic hemodynamic changes, pregnancy is also accompanied by numerous hormonal changes and metabolic adaptation. While most healthy individuals undergo these changes without complications, pregnancy can unmask or exacerbate underlying cardiovascular conditions, leading to serious maternal morbidity and mortality.

Over the last few decades, the reported maternal mortality rate (MMR) in the United States has been steadily increasing (9), in contrast to the declining rates observed among other high-income countries (10, 11). Part of this apparent rise in MMR in the United States since the early 2000s may reflect changes in surveillance methodology, particularly the implementation of the pregnancy checkbox on death certificates (12), which can increase the identification of maternal deaths, especially indirect obstetrical deaths (13). However, from 2018 to 2022, after full adoption of the pregnancy checkbox, the maternal mortality rate in the United States continued to increase from 17.4 to 22.3 deaths per 100,000 births, with a temporary spike to 32.9 deaths per 100,000 births in 2021, likely partly due to the COVID-19 pandemic (14). Moreover, while MMR captures maternal death during pregnancy and within 42 days postpartum, most pregnancy-related deaths occur between 42 days and 1 year postpartum (15). MMR in the United States also exhibits marked disparities by race and ethnicity, socioeconomic status, and geography. Non-Hispanic Black and American Indian/Alaska Native women experience a mortality rate that is at least two times higher than non-Hispanic White women (16). Cardiovascular diseases, including cardiomyopathy, coronary artery disease, hypertensive cardiovascular disease, and congenital heart diseases, are the leading cause of pregnancy-related deaths directly linked to pregnancy complications, especially among Black women (15, 16). Importantly, pregnancy complications, such as preeclampsia and gestational hypertension, are also strongly associated with long-term cardiovascular disease risk factors (17–21). These facts highlight an urgent need for a better understanding of cardiovascular complications during pregnancy.

**Figure 1:**
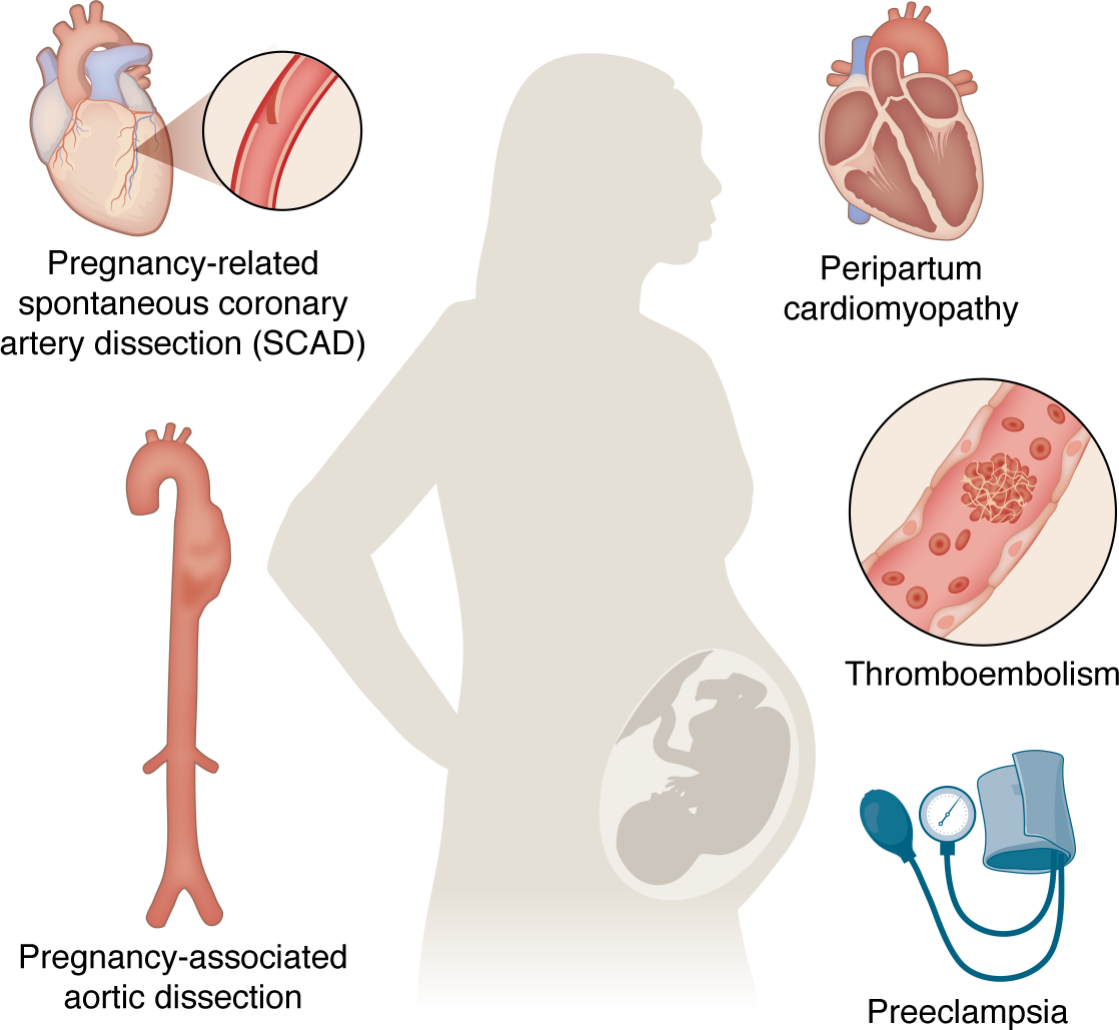
Cardiovascular complications of pregnancy. Cardiovascular diseases are the leading cause of pregnancy-related death in the United States.

This Review explores the physiological stresses of pregnancy, including hemodynamic, metabolic, vascular, hematologic, and renal adaptations. We also summarize the common acute cardiovascular complications that arise from the physiological stresses of pregnancy, such as preeclampsia, peripartum cardiomyopathy, pregnancy-associated aortic dissection, thromboembolism, and pregnancy-related spontaneous coronary artery dissection (SCAD) (Figure 1), and we explore the current understanding on their pathophysiology (Table 1). Congenital heart diseases and other preexisting cardiovascular diseases such arrhythmias, valvular disease, and chronic hypertension, also contribute to cardiovascular morbidity in pregnancy, but these preexisting conditions are beyond the scope of the current discussion and are covered in other comprehensive reviews (22–25).

## Physiology of cardiovascular changes during pregnancy

### Hemodynamic changes.

Hemodynamic changes during human pregnancy have been well characterized. The elevated hemodynamic burden starts in early pregnancy and peaks around 32 weeks of gestation. Plasma volume increases by 40%, with a milder increase in red blood cell volume, leading to an increase of total blood volume (26–29). Concurrently, systemic vascular resistance (SVR) decreases beginning in early pregnancy (5, 8, 30, 31), and blood pressure falls mildly, typically by about 5–8 mmHg (32–34). The regulation of SVR and blood pressure during gestation is complex. Vasodilation is in part driven by pregnancy-related hormones such as relaxin, progesterone, and estrogen (33, 35). At the same time, the volume-expanding and vasoconstrictive renal renin-angiotensin-aldosterone system (RAAS) is also activated, including elevated levels of angiotensin-converting enzyme (ACE), angiotensin (Ang) II, ACE2, and Ang-(1-7) (36). However, simultaneous resistance to this vasoconstrictive program also occurs, in part due to the development of insensitivity to Ang II (37–39) and due to the vasodilatory ACE II/Ang-(1-7) axis of the RAAS system (40). On balance, therefore, the activation of RAAS supports sodium and water retention and expands plasma volume while maintaining relatively low SVR and blood pressure. The subsequent increase in cardiac preload and decreased afterload, along with a concurrent rise in maternal heart rate, leads to a 15%–30% increase in stroke volume and a 20%–50% increase in cardiac output (CO), from roughly 4 L/min to 6 L/min (1–8, 30, 41, 42). The uterine artery flow reaches approximately 800 mL/min at term (43), accounting for approximately 10% of total maternal CO and roughly 40% of the gestational rise in CO. The remaining increase in CO is likely distributed to other maternal organs, underscoring that pregnancy induces systemic circulatory adaptations beyond the uteroplacental unit. Monochorionic twin pregnancies drive higher maternal CO, peaking earlier in the second trimester and likely driven by accelerated placental growth, but interestingly dichorionic twin pregnancies follow a more gradual increase akin to singleton pregnancies (44, 45).

### Cardiac structural adaptations.

In adaptation to this volume overload, left ventricle (LV) mass increases 10%–40% (7, 8, 46, 47), with an increase in LV end-diastolic and end-systolic chamber volumes and wall thickness, consistent with an eccentric hypertrophy phenotype (8). Animal studies demonstrate that the LV hypertrophy during pregnancy lacks cardiomyocyte cross-sectional hypertrophy, fibrosis accumulation, or fetal gene activation, distinguishing it from pathological hypertrophy induced by other insults (48–50). Cardiac function during pregnancy has been characterized both in human (2–4, 8, 51, 52) and animal models (48, 50, 53, 54), with variability observed within each. Studies with serial echocardiography performed throughout pregnancy have reported inconsistent findings on conventional cardiac function diameters such as ejection fraction, fractional shortening, velocity of circumferential fiber shortening (VcF_c_), and E/A ratio. While some report no meaningful change in systolic function during pregnancy (3, 8, 42), others suggest a mild reduction in cardiac contractility (2, 4, 36, 46, 48). In addition, several studies observed LV diastolic dysfunction and impaired myocardial relaxation in late pregnancy, which improves postpartum (2, 46, 47). Speckle-tracking echocardiography assesses subclinical changes in LV function and demonstrates a transient decrease in LV deformation (strain) in late pregnancy (8). Despite these variations, the common conclusion is that overall cardiac function remains within the normal range during pregnancy. Pregnancy-related changes in hemodynamic parameters and cardiac structures return to prepregnancy levels as early as 8 weeks postpartum in individuals with uncomplicated pregnancies (4, 41, 46). However, emerging evidence from small clinical studies suggest that in pregnancies with preexisting cardiovascular risk factors, such as obesity, there is a higher chance of persistent subtle cardiac diastolic dysfunction and impaired myocardial deformation (55, 56). Larger, well-controlled studies are needed to confirm these findings.

### Metabolism.

Pregnancy is accompanied with profound systemic metabolic changes to support the growing needs of the fetus, affecting metabolites including lipoproteins, fatty acids, amino acids, and glucose (57). These metabolic adaptations are dynamic, varying across trimesters and different maternal organs. Early pregnancy is considered an anabolic stage, gradually switching to a largely catabolic state at late pregnancy (1, 58). A major metabolic alteration during pregnancy is the development of insulin resistance. Despite this insulin resistance, by the third trimester, maternal circulating glucose levels drop, reflecting the large fetal consumption of glucose (58, 59). Increased insulin resistance also leads to increased lipolysis in adipose tissue, leading to higher triglycerides, LDL and HDL cholesterol levels in maternal circulation (57, 58, 60, 61), which peak and can reach atherogenic levels in late pregnancy (62). In the maternal heart, there is a fuel preference switch from glucose toward fatty acids during pregnancy, demonstrated largely in rodent models (63–65). This metabolic switch is PDK4 dependent and controlled by hormones such as progesterone and FGF21 (66, 67). Cardiac metabolic remodeling begins as early as the first trimester and persists throughout pregnancy (68). Although the heart can normally use a wide range of substrates to meet its high metabolic demands, fatty acids are highly favored during pregnancy, which may reduce cardiac energy efficiency and increase the vulnerability to pathological insult.

### Hematologic, vascular, and renal adaptations.

In addition to erythropoiesis, pregnancy also leads to a hypercoagulable state beginning in the first trimester, with greatly increased concentration and activity of procoagulant factors such as factor V, VII, VIII, X, von Willebrand factor, and fibrinogen, and decreased activity of anticoagulants such as antithrombin, fibrinolysis, and protein S (69, 70). This prothrombotic shift is largely driven by the sharp increase in estrogen and progesterone levels, which regulate the hepatic synthesis of procoagulant factors. The placenta also contributes antifibrinolytic influences by producing and secreting, from trophoblasts into maternal circulation, antifibrinolytic proteins such as plasminogen activator inhibitors 1 and 2 (71, 72). At the same time, the first-trimester placenta generates a localized profibrinolytic milieu through expression of tissue plasminogen activator, activated protein C, and other mediators (73, 74), likely important for placental growth but limited to the placenta. This dynamic balance between pro- and antifibrinolytic influences across pregnancy help ensure adequate uteroplacental blood flow while preventing excessive hemorrhage at delivery. However, these adaptations can also lead to increased risk of pregnancy-associated thromboembolic complications (75, 76).

Beyond the systemic vascular resistance change and RAAS activation discussed earlier, pregnancy also leads to additional vascular adaptations, most notably angiogenesis. Angiogenesis is particularly robust in the uterus during implantation and placental development, dramatically reducing vascular resistance in uteroplacental circulation, and thus shunting blood to the uterus and placenta. Pregnancy-associated angiogenesis also occurs in the maternal heart (49, 77), where increased vascular density may help accommodate the cardiac hypertrophy and enhanced workload.

Throughout pregnancy, the decrease in vascular resistance in kidney leads to an increase in renal plasma flow by 75% and glomerular filtration rate by 40%–50% (78, 79). Tubular handling of wastes and nutrients is also affected, leading to glucosuria, proteinuria, and decreased serum sodium levels (79–81). In addition to hydronephrosis, pregnancy also induces an increase in kidney volume (82), both of which contribute to kidney enlargement. Animal studies have also demonstrated an increase in kidney weight during pregnancy, but knowledge of the associated structural changes and molecular mechanisms remain limited. One study found early- to mid-pregnancy increases in the percentage of Bowman’s capsule in renal cortex (83). Another study suggested that structural changes that occur in the kidney during late pregnancy (E16 in mice) are prominent within renal medulla, with an increase in interstitial cellular constituents and cell proliferation in the glomeruli (84). Further studies are needed to better characterize the structural changes and molecular mechanisms underlying pregnancy-induced renal adaptations.

These physiological and systematic adaptations to pregnancy are necessary to maintain normal maternal function and support fetal development. However, the profound and complex changes in hormones, hemodynamics, metabolism, hematology, and vasculature during pregnancy also put women at higher risk for pregnancy-related complications. Importantly, these complications often arise from disturbances across multiple systems rather than a single pathway, reflecting the integrated nature of pregnancy physiology. While the contribution of individual factors to disease onset remains undefined, certain populations, including women with multiple gestations, advanced maternal age, or preexisting cardiovascular risk, are predictably more vulnerable. The specific mechanisms linking physiologic changes to adverse outcomes, and the populations at greatest risk, remain incompletely understood and represent important gaps in current knowledge, as outlined below.

## Cardiovascular complications of pregnancy

### Preeclampsia and gestational hypertension.

Preeclampsia and gestational hypertension (GH) are forms of new-onset hypertension that develop after 20 weeks of gestation, emerging in contrast to the normal trajectory of declining blood pressure during early pregnancy. Preeclampsia is distinguished from GH by the presence of proteinuria or other signs of end-organ damage, such as pulmonary edema, thrombocytopenia, or liver dysfunction (85). It is unclear whether preeclampsia and GH share the same etiology (86), although both conditions are associated with similar risk factors, including obesity, multiple pregnancy, and history of prior preeclamptic pregnancies.

Preeclampsia impacts 3%–8% of all pregnancies and is increasing in prevalence (87). Women who develop preeclampsia are at greater risk of maternal death, severe maternal morbidity, preterm birth, fetal growth restriction, and fetal and neonatal death (88). Most cases of preeclampsia are diagnosed near term (≥37 weeks’ gestation); the smaller proportion of cases diagnosed preterm are associated with greater maternal and fetal adverse outcomes. Severe forms of preeclampsia include HELLP syndrome, characterized by hemolysis, elevated liver enzymes, low platelet counts, and eclampsia, which is diagnosed in the setting of new-onset seizure. Notably, eclampsia may occur prior to the development of hypertension or other signs of preeclampsia. Twin pregnancies are associated with earlier onset and more severe manifestations of preeclampsia than singleton pregnancies (89).

The underlying pathogenesis of both preterm and term preeclampsia remains incompletely understood, but the placenta is thought to play a major role in the initiation of disease. Current understanding of the diseases comprises both placental and maternal dysfunction, with a central role of dysfunctional placental syncytiotrophoblasts (STBs), the major cellular interface between maternal and placental circulations (90). A “two-stage” model of preeclampsia proposes an initial stage of stressed STB that aberrantly secretes proinflammatory cytokines, reactive oxygen species, and antiangiogenic agents (such as soluble fms-like tyrosine kinase-1 [sFLT-1]) to the maternal circulation (91). These factors lead to a consequent stage of maternal maladaptation by promoting systemic inflammation, impaired vasodilation, and endothelial dysfunction (88, 90). Notably, the mechanisms of preeclampsia likely differ between preterm and term diseases, reflected in different anatomopathological featuresh of the placenta (92). Preterm preeclampsia is marked by malplacentation, which leads to placental malperfusion, causing STB dysfunction. Term preeclampsia, in contrast, is accompanied with fewer placental morphological changes (93). Proposed mechanisms for term preeclampsia include (a) the placenta outgrowing uterine capacity in late gestation, inducing STB stress (placental origin); and (b) preexisting cardiovascular risk factors such as obesity, or maternal genetic predisposition to hypertension (or preexisting hypertension), contributing to preeclampsia even with a healthy placenta (maternal origin) (94, 95). However, it remains unclear how risk factors such as obesity and chronic hypertension integrate into current pathogenic models, including whether they act through exacerbated hemodynamic load.

Low-dose aspirin has emerged as the only widely recommended pharmacologic intervention for the prevention of preeclampsia in high-risk women (34). Clinical trials have shown that aspirin, when initiated before 16 weeks of gestation, can reduce the incidence of preterm preeclampsia and related adverse outcomes (96), likely by modulating prostacyclin-thromboxane balance and its downstream effects on placentation, angiogenesis and inflammation. Nevertheless, aspirin prophylaxis does not fully eliminate risk, particularly for later-onset disease. Continued development and refinement of animal models are essential to dissect pathogenic mechanisms and identify additional therapeutic targets. But the complexity and heterogeneity of the diseases make it challenging to develop a reliable animal model to fully recapitulate the disease. Table 2 summarizes validated animal models currently used to study the pathophysiology of preeclampsia.

In summary, various animal models greatly contribute to the understanding of the diseases, including pathophysiology, predictive biomarkers, novel therapies, and long-term cardiovascular effects. However, none of the current animal models fully recapitulate human preeclampsia and they are limited by (a) the fact that very few animal models develop severe phenotypes such as HELLP and eclampsia (97, 98); (b) model animals such as rodents have distinct reproductive system anatomy and pregnancy physiology, with for example more shallow placentation than humans, or lacking the expression of all the isoforms of sFLT-1 that are expressed in human; (c) most animal studies do not replicate the primary event of abnormal placentation, thus limiting the ability to test potential therapies targeting early placentation; and (d) additional confounding factors such as multiparous pregnancies in rodents and variable fetal genotypes in genetic models, which add complexity and limit generalizability.

### Peripartum cardiomyopathy.

Peripartum cardiomyopathy (PPCM) is an uncommon but potentially fatal cardiovascular complication of pregnancy. PPCM is diagnosed in the setting of heart failure with LV systolic dysfunction (LV ejection fraction <45%) that occurs towards the end of pregnancy or within several months after delivery, in the absence of other known cause of heart failure (99). Most commonly, PPCM is diagnosed within the first week of delivery, although delayed diagnosis is common, especially among Black women (100). PPCM occurs in an estimated 1 in 2,000 births, although regional variation exists. At least one-third of cases of PPCM are accompanied by preeclampsia, and risk factors for PPCM are similar to those for preeclampsia, suggesting common pathogenesis. In a prospective North American registry of 100 patients with PPCM, the majority recovered myocardial function after 1 year, but 13% experienced death, heart transplant, or persistent severe cardiomyopathy (101).

The etiology of PPCM remains poorly understood. There are several hypothesized mechanisms of PPCM, including (a) hemodynamic stress, (b) viral myocarditis, (c) nutrition deficiency, (d) autoimmune disease, and (e) hormonal factors. The first of these, i.e., that the heart fails to respond to the stress of dramatic hemodynamic changes during pregnancy or that PPCM is an exacerbation of the mild cardiac dysfunction in late pregnancy (102), fits poorly with the typical timing of PPCM presentation after delivery, when the hemodynamic parameters are returning to baseline level. Viral myocarditis was proposed in the 1990s upon finding histological myocarditis in 78% of PPCM biopsies from 18 patients (103), but subsequent studies have yielded conflicting results, reporting a much lower incidence of myocarditis (104), and similar incidence of virus genomes in both PPCM and healthy control biopsies (105, 106). Modern techniques using cardiovascular magnetic resonance and late gadolinium enhancement (LGE) imaging to evaluate myocarditis in PPCM patients have reported incidences that vary dramatically, from as high as 77% (107) to as low as 7.5% (108). This striking disparity in both histology and LGE findings raises doubt on the consistency and validity of myocarditis as a central pathogenic mechanism in PPCM. Nutrition deficiency, specifically selenium, has been suggested as a possible risk and therapeutic target for PPCM in certain parts of Africa (109, 110). However, selenium supplementation failed to show therapeutic benefit in PPCM patients in Haiti (111). The hypothesis that PPCM is an organ-specific autoimmune response due to defective fetal-placental allograft tolerance during pregnancy is based on findings that PPCM patients have high titers of autoantibodies and altered immune profile (112). Additionally, a 1999 study showed that the administration of immunoglobulin, an immunomodulator, improved cardiac systolic function in PPCM patients (113), but no further studies have been reported.

**Figure 2:**
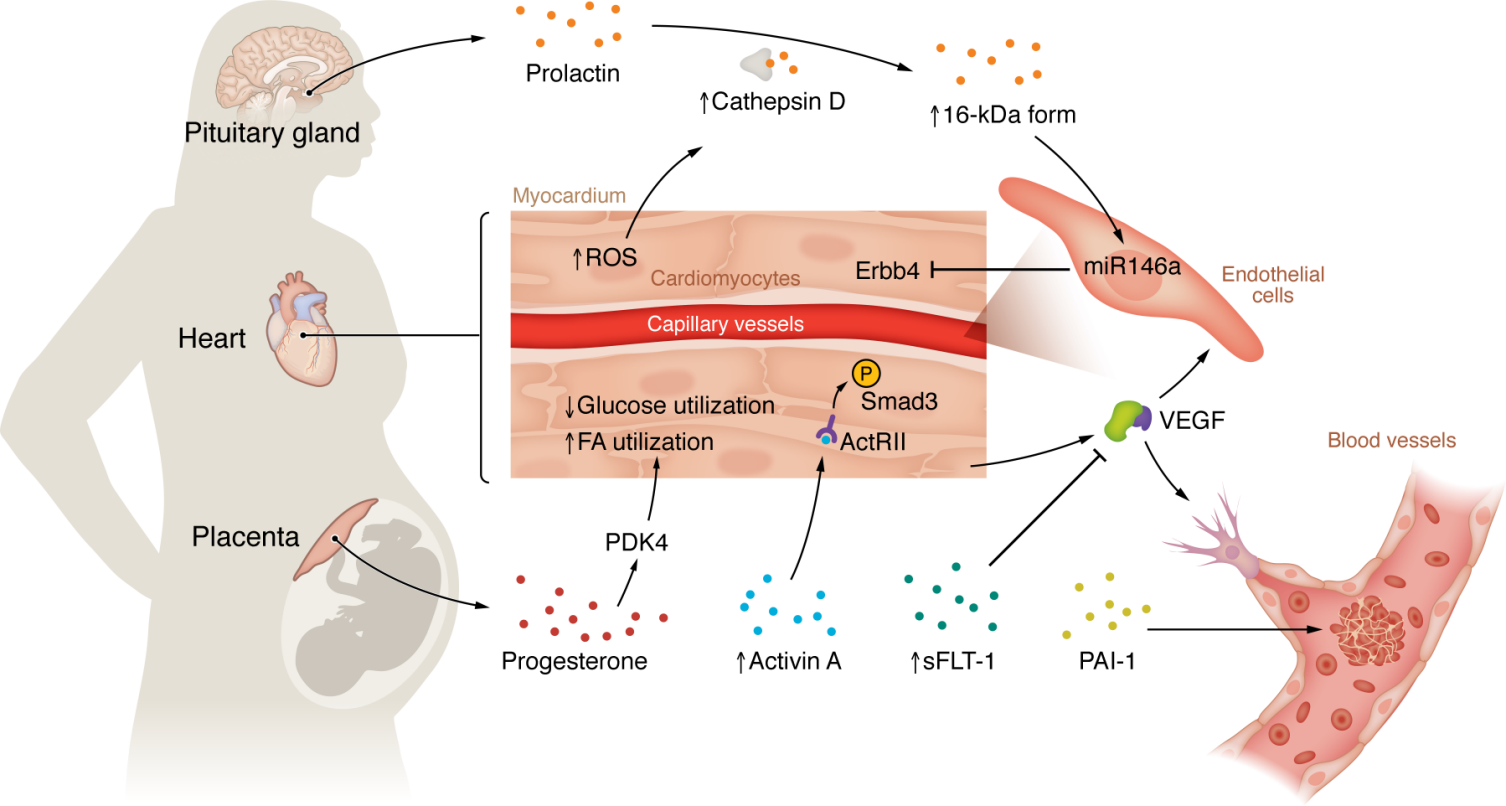
Mechanisms of pregnancy-related cardiovascular complications. Factors produced by the brain and placenta during pregnancy lead to cardiovascular remodeling, which can result in the development of pregnancy-associated cardiovascular disease. ActRII, activin receptor type II; PAI-1, plasminogen activator inhibitor-1; PDK4, pyruvate dehydrogenase kinase 4; sFLT-1, soluble Fms-like tyrosine kinase 1.

More recent studies have elucidated the role of pregnancy-specific hormones in the pathogenesis of PPCM. PPCM patients with end-stage heart failure had reduced cardiac expression of STAT3, a transcription factor with broad functions, and a cardiac-specific STAT3-knockout mouse model developed PPCM (77). Mechanistically, loss of STAT3 in this model enhanced oxidative stress during pregnancy, leading to increased activity of cathepsin D, which cleaves the pregnancy hormone prolactin into its antiangiogenic 16-kDa form and leads to microvascular deficiency (77). Another cardiac-specific knockout model, in this case of the transcription regulator PGC-1α, also developed PPCM. In this model, PGC-1α deficiency reduced expression of VEGF, a critical angiogenic factor, which was further neutralized by placenta-derived sFLT-1, a potent inhibitor of VEGF, thereby again leading to microvascular deficiency (114). Notably, this mechanism may explain the strong epidemiologic association between preeclampsia and PPCM. Further research is ongoing to understand the pathophysiological roles of other placenta-derived hormones. A recent study found that blocking the receptor of activin A, a hormone that is greatly increased in sera from patients with preeclampsia and PPCM, improves cardiac function in the PGC-1α model of PPCM (115, 116), likely through the inhibition of downstream Smad3 signaling (117) (Figure 2).

Finally, it has also become apparent that strong genetic predispositions to PPCM exist in at least a subset of women. Women with PPCM have a substantially higher prevalence of loss-of-function variants in several genes, including *TTN*, *FLNC*, *DSP*, and *BAG3* (118). Interestingly, the prevalence of these variants in women with PPCM is nearly identical to that found in patients with idiopathic dilated cardiomyopathy, indicating common pathomechanisms of these two diseases. How these variants interact with pregnancy hormones to cause disease remains unclear. Moreover, fewer than 25% of women with PPCM have identifiable variants. Thus, much remains to be learned of how PPCM develops.

### Acute myocardial infarction/SCAD.

Although rare, pregnancy is associated with a 4-fold higher risk of myocardial infarction (MI) compared with reproductive-age women who are not pregnant (119). Pregnancy-associated MI may be caused by atherosclerosis, SCAD, or other causes, such as in situ thrombosis or embolism (120). SCAD in general disproportionately affects women and has been estimated to cause up to 43% of pregnancy-associated MI (121). Like PPCM, SCAD is most likely to occur in the early weeks after delivery (122). SCAD is a nonatherosclerotic cause of acute MI that is caused by intramural hematoma in the epicardial coronary artery, with or without intimal tear (123). Pregnancy-associated SCAD is associated with a worse prognosis compared with SCAD that occurs not in the setting of pregnancy, with larger infarcts and lower LV ejection fraction (124) that frequently result in maternal death (125). Due to the strong sex disparity in SCAD, and its pregnancy susceptibility, several hypotheses of mechanism of disease center on sex hormone association, including estrogen and progesterone withdrawal (126), and prolactin level elevation (127). However, evidence supporting these associations remains limited. Although the pathophysiology of SCAD is not well understood, fibromuscular dysplasia is diagnosed in more than half of all patients with SCAD (121).

Genetic predispositions to SCAD have also been explored in recent years. Several genes involved in vascular connective tissue integrity have been implicated, such as *COL3A1* and *COL4A1*, which encode fibrillar collagens known to be associated with vascular connective tissue disorders and arterial rupture (128). Notably, mice with Col3a1 deficiency (*Col3a1*^+/–^) exhibit aortic root enlargement, with more pronounced effects in female mice, which can be further exacerbated by pregnancy, aligning with human epidemiology (128). A genome-wide association study involving 85 patients with pregnancy-associated SCAD (P-SCAD) identified actin filament-associated protein AFAP1 at chromosome 4q16.1 to be specifically associated with the disease (127). A subsequent study also linked AFAP1 to SCAD more broadly (129). These findings highlight that while genetic predisposition plays a role, it likely acts in concert with pregnancy-related stresses in the pathogenesis of P-SCAD.

### Venous and arterial thromboembolic diseases.

The hypercoagulable state of pregnancy likely serves to maintain placental function and meet hemostatic requirement during labor, and lasts until 8 weeks postpartum, in part as a result of the expulsion of the placenta at term (130). This pregnancy-associated hemostatic environment puts women at 3- to 5-fold higher risk of overall thromboembolism, approximately 80% of which are venous (VTE, such as deep venous thrombosis, pulmonary embolism) and 20% are arterial (stroke and heart attack) (75, 119, 131). The increased risk of thromboembolism increases with gestation age, from 2-fold at early pregnancy, to 9-fold at late pregnancy, and reaches 80-fold within 6 weeks postpartum (132). There are several risk factors that contribute to VTE in the nonpregnant population, such as aging, obesity, immobilization, recent travel, etc. Interestingly, the prevalence of these preexisting risk factors in pregnancy-associated VTE are lower than in nonpregnancy VTE based on data from an international VTE registry (133). In contrast, pregnancy-specific risk factors, such as cesarean delivery, preeclampsia, postpartum infection, and in vitro fertilization, are associated with increased risk of peripartum thromboembolic events (132, 134). Women with inherited thrombophilia and autoimmune diseases, such as antiphospholipid syndrome, are also at increased risk for thromboembolism and other adverse pregnancy outcomes, including recurrent pregnant loss (135).

### Aortic dissection.

Aortic dissection (AoD) is a medical emergency that involves the avulsion of the intimal layer of the aortic wall, leading to the formation of a “false lumen” that reroutes arterial blood. AoD typically affects people over 65 years old and is more common in men than in women. Inherited connective tissue disorders such as Marfan syndrome, Loeys-Dietz syndrome, and Ehlers-Danlos syndrome are well-known precursors of AoD due to the disruption of intimal extracellular matrix and loss of smooth muscle cells. Hypertension also contributes to AoD potentially by directly applying pressure to the aortic wall and creating a proinflammatory milieu in the vasculature (136).

Pregnancy-associated AoD (P-AoD) is rare, with an incidence of 0.55–0.69 among 100,000 pregnancies based on studies performed in the United States (137, 138). Pregnancy is associated with a higher risk of AoD, with the highest risk seen among pregnant women with connective tissue or hypertension disorders (137, 138). The time of onset of P-AoD varies, with some studies concluding that the majority (58.8%–77.9%) of P-AoD happens in the third trimester (138, 139), while others suggest that more patients develop P-AoD postpartum (140). The mortality rate of P-AoD ranges from 3% to 23% (138–140), with variability likely due to a relatively small number of cases in most cohorts (137, 138, 140), and difference in the types of AoD cases (i.e., type A, which involves the ascending aorta and has a higher mortality rate, vs. type B, which involves the descending aorta), and variable prevalence of connective tissue disorders.

Two studies from the same group, using animal models of Marfan syndrome (*Fbn1*-deficient mice) and vascular Ehlers-Danlos syndrome (*Col3a1* mutation–knockin mice), identified lactation as a major contributor to the occurrence of P-AoD (141, 142). In both models, lactation increased 30-day postpartum lethality due to arterial dissection or rupture compared with virgin female mice (91.1% vs. 6.7% in *Fbn1*-deficient mice; 54% vs. 4% in *Col3a1*-mutant mice). Either pup removal or treatment with oxytocin receptor antagonist effectively rescued postpartum death due to P-AoD in both studies. While there is currently insufficient evidence to determine whether these findings in mice are applicable to human patients, future clinical studies may benefit from including lactation history when assessing P-AoD risk.

### Other complications with cardiovascular implications.

Gestational diabetes mellitus (GDM) is primarily a metabolic disorder characterized by impaired glucose tolerance first recognized during pregnancy. While not a cardiovascular disease per se, GDM has important implications for maternal cardiovascular health. Over the past few decades, accumulating evidence has shown that GDM increases the risk of future diabetes and cardiovascular disease (143). More recent studies suggest that women with GDM may develop subclinical cardiac symptoms during pregnancy, with increased cardiac mass and impaired cardiac energetics and contractility (144). Importantly, these subclinical changes can persist into the postpartum period, partially independent of progression to overt type 2 diabetes (145). Although the mechanisms are not well studied, one potential mechanism is altered cardiac metabolism, where shifts in substrate utilization may contribute to impaired cardiac energetics (50). This remains a relatively understudied area, and given the rising prevalence of obesity and GDM, larger and more comprehensive clinical and animal studies are needed to clarify these metabolic-cardiac interactions and their long-term consequences.

## Racial and ethnic disparities

Based on national data from 2022, the Unites States continues to have a higher rate of maternal deaths compared with other high-income countries (146). Within the Unites States, marked racial disparities exist; non-Hispanic Black women have a 2.5-fold greater risk of maternal mortality compared with non-Hispanic White women (14). Based on data published from state-based maternal mortality review committees between 2017 and 2019, the most common cause of pregnancy-related deaths, which are deaths that occur during pregnancy or the 12 months following delivery, also differed substantially by race. Mental health conditions, including suicide and substance use disorder, were the leading causes of death in White women, whereas cardiac conditions and cardiomyopathy were the leading causes of death in Black women (15). Black women have a higher prevalence of PPCM compared with White women, are diagnosed with more severe disease, and experience lower rates of recovery (147). In the Investigations of Pregnancy-Associated Cardiomyopathy (IPAC) cohort, 26% of Black women had a major adverse event, including death, transplant, or persistent severe cardiomyopathy compared with 8% of non-Black women (*P* = 0.03) (101). Deaths from preeclampsia are 2–3 times higher in Black compared with White women (148). Among women with pregnancy-induced hypertension, Black women have a 2-fold higher risk of stroke compared with White women (149). Racial differences have also been reported for pregnancy-associated MI. The underlying causes of these disparities are only partially explained by higher rates of chronic medical conditions experienced by Black individuals. Genetic predispositions may also play a role. Sickle cell trait, which is highly prevalent among Black individuals, has been linked to adverse pregnancy outcomes, including preeclampsia, VTE, and preterm delivery (150). And fetal *APOL1* variants, which are found almost exclusively in individuals of African ancestry, strongly link to preeclampsia and other adverse pregnancy outcomes (151), and forced placental expression of these *APOL1* variants causes preeclampsia in mice (152). Beyond biologic predispositions, differences in access to care and quality of care prior to pregnancy and in the peripartum period likely contribute to racial disparities in cardiovascular outcomes. For example, in New York City, up to 48% of racial disparities in severe maternal morbidity were explained by differences in delivery hospital location (153). Social determinants of health also impact disparities in maternal cardiovascular outcomes through social, environmental, economic, and biologic mechanisms (154, 155).

## Conclusions and future directions

Pregnancy induces profound physiological changes to the maternal cardiovascular system. While most women adapt to these changes, a subset experience acute cardiovascular complications that contribute strongly to maternal morbidity and mortality. Pregnancy-induced cardiovascular complications during pregnancy — such as preeclampsia, PPCM, AoD, and thromboembolism — reflect complex interactions between genetic predisposition, hormonal signaling, metabolic adaptation, and vascular remodeling. These complications impact not only maternal and fetal outcomes during pregnancy, but also contribute to short-term postpartum morbidity and long-term cardiovascular risks later in life. Despite a steadily improving epidemiological understanding of these diseases, and an increasing number of preclinical models to study them, there remain few to no disease-specific treatments for any of these pregnancy complications. New challenges are also emerging, particularly the rising prevalence of obesity and metabolic disorders in women of childbearing age. In this context, the use of incretin-modifying drugs (e.g., GLP-1 receptor agonists) prior to conception, and often unknowingly during early gestation, and the metabolic rebound following their discontinuation, raises important unanswered questions. These agents not only affect systemic metabolism but also exert direct beneficial effects on the cardiovascular system, making their impact on pregnancy physiology and outcomes a critical area for future investigation. Advancing our understanding of the pathophysiology underlying cardiovascular complications of pregnancy, as well as their social determinants, is our best hope to improving pregnancy outcomes and informing preventive strategies for high-risk populations. Progress in this area critically depends on animal models, given the inherent limitations of conducting mechanistic studies in pregnant humans. While organoid and in vitro systems provide valuable insights into placental or vascular biology, they cannot recapitulate the complex, integrated maternal adaptations involving hemodynamic, metabolic, vascular, and hormonal changes. Thus, animal models remain irreplaceable tools for dissecting pregnancy physiology and pathology, and there is an urgent need to expand and diversify their use to better capture disease complexity and accelerate the discovery of effective therapies.

## Funding support

This work is the result of NIH funding, in whole or in part, and is subject to the NIH Public Access Policy. Through acceptance of this federal funding, the NIH has been given a right to make the work publicly available in PubMed Central.

- American Diabetes Association fellowship 1-25-PDF-106 (to YY).
- NIH grants HL173331 (to JL) and HL152446 (to ZA).

## Figures and Tables

**Table 2:**
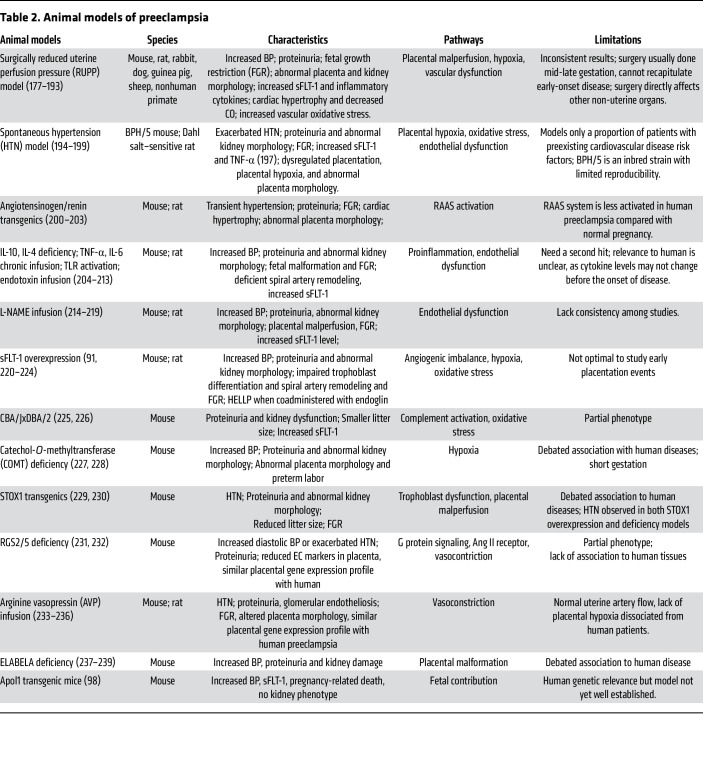
Animal models of preeclampsia

**Table 1:**
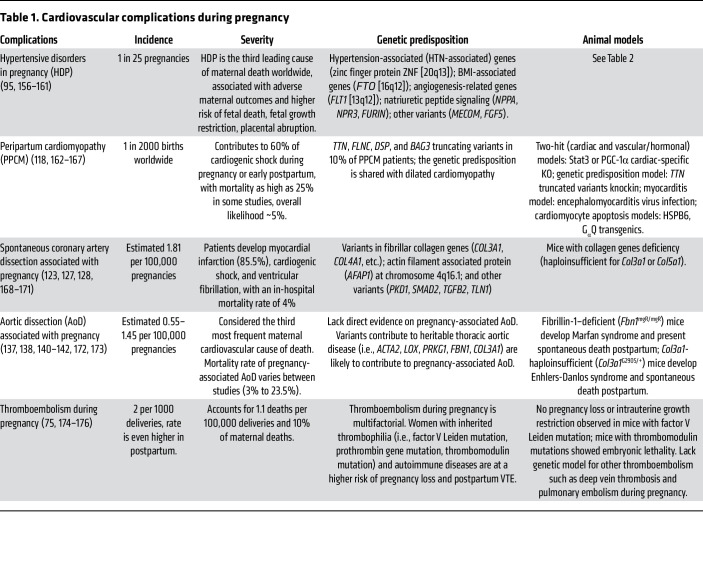
Cardiovascular complications during pregnancy

## Version 1 01/02/2026

Electronic publication
